# Correction of a Splicing Mutation Affecting an Unverricht-Lundborg Disease Patient by Antisense Therapy

**DOI:** 10.3390/genes9090455

**Published:** 2018-09-11

**Authors:** Liliana Matos, Ana Joana Duarte, Diogo Ribeiro, João Chaves, Olga Amaral, Sandra Alves

**Affiliations:** 1 Research and Development Unit, Department of Human Genetics, INSA, 4000-055 Porto, Portugal; liliana.matos@insa.min-saude.pt (L.M.); ana.duarte@insa.min-saude.pt (A.J.D.); diogo.ribeiro@insa.min-saude.pt (D.R.); olga.amaral@insa.min-saude.pt (O.A.); 2 Neurology Service, Santo António Hospital, CHP, 4099-001 Porto, Portugal; joaomchaves71@hotmail.com

**Keywords:** antisense oligonucleotides, *CSTB* gene, progressive myoclonic epilepsy type 1, splicing mutation, splicing therapies

## Abstract

Unverricht-Lundborg disease (ULD) is a common form of progressive myoclonic epilepsy caused by mutations in the cystatin B gene (*CSTB*) that encodes an inhibitor of several lysosomal cathepsins. Presently, only pharmacological treatment and psychosocial support are available for ULD patients. To overcome the pathogenic effect of the ULD splicing mutation c.66G>A (exon 1), we investigated whether an antisense oligonucleotide therapeutic strategy could correct the defect in patient cells. A specific locked nucleic acid (LNA) antisense oligonucleotide was designed to block a cryptic 5′ss in intron 1. Overall, this approach allowed the restoration of the normal splicing pattern. Furthermore, the recovery was both sequence and dose-specific. In general, this work provides a proof of principle on the correction of a *CSTB* gene defect causing ULD through a mutation-specific antisense therapy. It adds evidence to the feasibility of this approach, joining the many studies that are paving the way for translating antisense technology into the clinical practice. The insights detailed herein make mutation-based therapy a clear candidate for personalized treatment of ULD patients, encouraging similar investigations into other genetic diseases.

## 1. Introduction

Unverricht-Lundborg disease (ULD), also known as progressive myoclonic epilepsy type 1 (EPM1), is an autosomal recessive neurodegenerative disorder characterized by young-onset stimulus sensitive myoclonus and tonic–clonic seizures [1]. Although this disease is worldwide, it is most prevalent in Finland, North Africa and the Western Mediterranean region [2]. Presently, symptomatic pharmacologic and rehabilitative management are the mainstays of patient care [2,3]. These patients are often difficult to diagnose, and multiple attempts are made, which can amount to several years of suffering, multitudes of exams, misdiagnosis, and blind attempts to carry out symptomatic treatments.

Unverricht-Lundborg disease is caused by mutations in the *CSTB* gene, localized on chromosome 21q22.3 [4], which encodes cystatin B (CSTB): a protease inhibitor that is able to inhibit several lysosomal cysteine proteases in vitro by reversible tight-binding. Those proteases are known as cathepsins [5].

Because CSTB is ubiquitously expressed and functions in healthy cells as an inhibitor of the cathepsin family of proteases, it is not surprising that loss of CSTB activity affects a broad range of cellular biological functions, including: neuronal death, redox homeostasis, hyperexcitability, and glial activation [6]. Up to now, 15 *CSTB* mutations have been described as causal of ULD (HGMD^®^Professional 2018.1 Release), the most common of which is an unstable expansion of a dodecamer repeat in the promoter region [7] that down-regulates *CSTB* mRNA levels [8]. The remaining mutations are missense, nonsense, frameshift and splice-site (ss) mutations, leading to abnormal RNA processing (HGMD^®^Professional 2018.1 Release).

At present, the increasing knowledge on RNA biology is encouraging new approaches of therapeutic RNA-based strategies to modify or eliminate an mRNA bearing a disease-causing mutation. Antisense oligonucleotide (AO)-based therapies are emerging as an efficient alternative for drug development that can be employed in future as a new treatment for a number of diseases. Currently, several therapeutic strategies using AOs are being developed and applied to a number of diseases, including rare genetic disorders such as Duchenne muscular dystrophy [9,10,11], spinal muscular atrophy [12,13,14], Hutchinson-Gilford progeria syndrome [15], pyridoxine dependent epilepsy [16], and several other common neurodegenerative diseases such as Alzheimer’s [17], Parkinson’s [18], and Huntington’s [19], among others [20,21].

Recently, our group described a Portuguese ULD patient who is homozygous for a new synonymous mutation (c.66G>A; p.Q22Q) which leads to mis-splicing of *CSTB* pre-mRNA, and, subsequently reported the mislocalization of the resulting mutant protein [22,23]. Two transcripts were found in the patient-derived fibroblasts: a normal transcript with the synonymous G>A change at the last nucleotide of exon 1, and a mutant one including 354 bp of intron 1 due to the activation of a cryptic 5′ss, which is expected to result in the production of an abnormal peptide with a premature truncation. In this work, we have exploited an AO approach to correct the splice defect associated with the c.66G>A splice-site mutation in patient cells. We report that the use of a specific locked nucleic acid (LNA) AO designed to block the activated cryptic splice site in intron 1, succeeded in restoring the normal splicing pattern. In fact, only the normal transcript was produced after treatment.

Given that the present treatments for ULD are only symptomatic, these results suggest that splice-modifying AOs might be a potential alternative or adjunct treatment strategy for patients holding splicing changes in the *CSTB* gene. As far as we know, this is the first report on a patient-tailored therapeutic correction in cells from an individual with ULD.

## 2. Materials and Methods

### 2.1. Biological Material

In a study where the mutation effects at mRNA and protein levels were analyzed [22], a Portuguese patient with a clinical picture compatible with ULD was previously reported to be homozygous for the c.66G>A change in the *CSTB* gene. Skin fibroblasts were obtained from both the patient and a healthy control with appropriate informed consent. The study was conducted in accordance with the Declaration of Helsinki, and the protocol was approved by the Ethics Committee of Instituto Nacional de Saúde Dr. Ricardo Jorge (2015DGH1062). Confidentiality of personal data for all samples was protected. Both fibroblast cell lines were cultured following standard procedures in DMEM medium (Gibco Invitrogen, Carlsbad, CA, USA) with 10% Fetal Bovine Serum (FBS) and 1% kanamycin at 37 °C and 5% CO_2_.

### 2.2. Cycloheximide Treatment of Control and Patient Fibroblasts

To perform the nonsense-mediated mRNA decay (NMD) assays, both fibroblast cell lines were cultured in the presence of two different concentrations of cycloheximide (100 and 600 µg/mL) for 8 h. Total RNA was then isolated using the High Pure RNA Isolation kit (Roche Applied Science, Indianapolis, IN, USA) and reverse-transcribed using the “Ready-To-Go You-Prime First-Strand Beads” kit (GE Healthcare, Buckinghamshire, UK) adhering to the manufacturer’s protocol. Specific primers for exon 1 (Fw: 5′-GCCGAGACCCAGCACATC-3′) and exon 2 (Rv: 5′-TGACACGGCCTTAAACACAG-3′) were used to amplify complementary DNA (cDNA) fragments encompassing the wild-type (WT) and the mutated region, where the *CSTB* gene splicing was altered as described in Pinto et al. [22]. After reverse transcription (RT)-PCR amplification and electrophoretic separation, the obtained bands were cut and purified using the Wizard^®^ SV Gel and PCR clean-up system (Promega, Madison, WI, USA), and then sequenced.

### 2.3. Antisense Oligonucleotide Treatment and Analysis

The LNA oligonucleotide targeted to the cryptic donor splice site in the *CSTB* intron 1 was designed, synthesized and purified by Exiqon (Vedbaek, Denmark). The LNA sequence is 5′-AGCCGGCTGCTCACCTGCGCCATCGCCG-3′. A standard LNA control oligonucleotide (also provided by Exiqon) was used as a negative control. Its sequence is: 5′-TAACACGTCTATACGCCCATAACACGTCTATACGCCCATAACACGTCTAT-3′. For the LNA treatment, between 2.5 and 3.5 × 10^5^ fibroblast cells were grown in 6-well plates, and after 16 h treated with different concentrations of LNA (5, 25, 50 and 100 nM) using Lipofectamine LTX (Invitrogen, Carlsbad, CA, USA) as the delivery reagent. Cells were harvested 24 h later. Total RNA was isolated and cDNA synthesized as described above. The RT-PCR analysis of the patient-derived transcripts was performed using the above described primers.

### 2.4. CSTB Protein Immunodetection

For Western blot analysis, between 1.4 and 1.6 × 10^6^ patient fibroblast cells grown in 75 cm^2^ flasks were transfected with 100 nM of LNA using Lipofectamine LTX (Invitrogen, Carlsbad, CA, USA), and harvested after 24 h. Total protein extracts, obtained from patient fibroblasts with and without LNA treatment, as well as from a healthy control sample, were homogenized in a lysis buffer solution (supplemented with a protease inhibitor cocktail) and ruptured by three cycles of 5-min freeze-thawing, and then briefly centrifuged. Protein concentration was determined, measuring the absorbance at 280 nm in a NanoDrop^®^ND-1000 Spectrophotometer (Thermo Fisher Scientific, DE, USA). Equal amounts of total protein crude extracts were loaded on a 4%–12% NuPAGE^®^Novex^®^Bis-Tris precast gel (Invitrogen, Carlsbad, CA, USA). After electrophoresis, proteins were transferred to a nitrocellulose membrane in a semi-dry transfer system for 2 h at 150 mA. Ponceau staining was used to monitor equal loading of protein. Immunodetection was carried out using anti-Stefin B antibody (mouse monoclonal IgG1 reacting with human Stefin B: ab54566 from Abcam—Cambridge Science Park, Cambridge, UK) followed by an incubation with goat anti-mouse IgG-HRP as the secondary antibody (conjugated goat anti-mouse IgG horseradish peroxidase: sc-2005, Santa Cruz Biotechnology, TX, USA). The total amount of protein loaded was controlled by incubation with monoclonal anti-α-tubulin antibody (T6199—Sigma-Aldrich, St. Gallen, Switzerland). The protein signal was developed using the Enhanced Chemiluminescence System (GE Healthcare, Buckinghamshire, UK).

## 3. Results

### 3.1. The CSTB c.66G>A Mutation Generates One Aberrant Transcript in Unverricht-Lundborg Disease Patient

The *CSTB* transcription profile was obtained by RT-PCR, and showed in patient fibroblasts both an aberrant and a normal transcript. In healthy control cells, only the normal transcript was observed. To determine whether the expression level of aberrant transcript/s were altered by NMD, control and patient fibroblasts were treated with either 100 or 600 µg/mL cycloheximide, but no extra band or increased expression were observed besides the described transcripts (Figure 1). Direct sequencing of the bands confirmed the sequence pattern originally described in Pinto et al. [22]. These experiments indicated that the *CSTB* c.66G>A mutation generates one aberrant transcript that is not degraded by NMD.

### 3.2. Correction of *CSTB* Mutation-Induced Missplicing Using an Locked Nucleic Acid Oligonucleotide in Patient-Derived Fibroblasts

As an approach to correct *CSTB* exon 1 splicing, a specific 28-mer LNA oligonucleotide was used to block the activated cryptic 5′ss within intron 1 (Figure 2A). Different quantities of the LNA were transfected into patient fibroblasts, and the endogenously spliced *CSTB* transcripts from 24 h treated cells were then submitted to RT-PCR. The cDNA pattern showed that treatment with increasing amounts of LNA abolished the aberrantly spliced transcript with the insertion of 354 bp of intron 1 in a dose-dependent manner, with total correction being achieved at 100 nM of LNA (Figure 2B). The LNA treatment proved to be sequence-specific, since a LNA with a scrambled sequence has no effect on the exon 1 splicing pattern in patient fibroblasts (data not shown).

In order to analyze whether the observed splicing correction led to restoration of CSTB protein levels in fibroblasts, the protein was detected by Western blot after treatment of control and patient cell lines with 100 nM of the specific LNA. The Western blot protein analysis (Figure 2C) revealed the presence of a band with 11 kDa, which corresponds to the normal CSTB protein in control and patient fibroblasts (treated and untreated with LNA). Therefore, the treatment does not seem to interfere with CSTB protein expression. The abnormal truncated protein is not detected in patient fibroblasts, suggesting low amounts of protein below the detection limit of the antibody.

## 4. Discussion

RNA missplicing diseases account for up to 15% of all inherited diseases, including neurological, myogenic and metabolic disorders [24].

Mutations affecting splicing have often been neglected, either because they appear to be silent synonymous changes with no effect on amino acid sequence, or due to their inconspicuous intronic location. However, with the increasing knowledge arising from fine cDNA analysis and genomic sequencing of patients suffering from a wide range of diseases, the number of known exonic or intronic mutations that affect splicing has increased to 50%–60% of all annotated disease-causing mutations [25,26]. It is therefore understandable that during the last decade, genetic therapy directed toward correction of RNA mis-splicing has progressed from theoretical work in cultured cells to promising clinical trials [24,27,28]. In this work, we have developed and tested an AO approach, in order to investigate the possible mitigation of the splice defect present in a ULD Portuguese patient. Modified AOs that hybridize by complementarity to a selected site in the pre-mRNA have been used to redirect splicing, allowing restoration of the gene function [27,29,30].

The ULD patient here addressed was homozygous for c.66G>A, a mutation that does not alter the coding amino acid sequence (p. Q22Q), but instead activates a cryptic splice site downstream in *CSTB* intron 1, probably because its presence weakens the recognition of the normal splice site by the U1. An aberrant transcript with the inclusion of 354 bp of intron 1 is produced that was found to avoid NMD mRNA decay, since the intensity of the expressed transcript did not increase after treatment with cycloheximide—a potent inhibitor of the mechanism (Figure 1). Once the transcript is not degraded, it is predictable that it will give rise to a truncated protein with only 36 amino acids, 13 of which are not present in the normal CSTB protein that is made up of 98 residues. At least in the patient fibroblasts, the normal transcript is also generated and normal CSTB protein is expressed, although at lower levels than in control cells (Figure 2C). This raises the possibility that the cause of the disease in the patient could be the combined effect of the reduced levels of the CSTB protein and the production of a truncated protein that possibly exerts a toxic or dominant negative role [23], as was evidenced for other CSTB mutant proteins [31,32]. It is described that *CSTB* mutants that affect protein sequence are prone to aggregate in cells, even though it is unclear whether protein misfolding and aggregation is responsible for augmenting progression of the disease and neurodegenerative changes, or whether it is the lack of the protein’s function, or a combination of both [31]. It can be speculated that CSTB proteins can indeed modulate disease phenotype through a toxic gain of function mechanism [31,32]. Genotypes associated with low CSTB protein levels, such as compound heterozygosity of the dodecamer expansion with a point or indel mutation, tend to be associated with more severe clinical features than the classic dodecamerhomozigosity [33,34,35], which reinforces that mutant CSTB proteins can indeed lead to modulation of the disease phenotype. Whatever the case, we strongly believe that an approach, that is able to promote the elimination of the aberrant transcript and, consequently, of its resulting abnormal protein, such as the one we here present, may represent a key step toward a personalized therapeutic correction for this patient.

As a strategy to suppress the splicing defect, we used a specific LNA oligonucleotide to block the activated cryptic splice site in intron 1, which effectively abolished the aberrant splicing process of *CSTB* pre-mRNA in patient-derived cells. The normal splicing pattern leading to a single transcript with the synonymous change G>A was successfully rescued, and so the expected corrective effect was achieved. Furthermore, the recovery was sequence and dose-specific without noticeable evidence of increased cell death after treatment in vitro (Figure 2B). In this way, this work adds evidence to the feasibility of antisense therapy, joining the many studies that are paving the way for translating the technology into the clinical practice [36,37,38,39,40].

By now, a major challenge is the safe and targeted delivery of AOs to specific tissues—an issue that hopefully will be overcome in the near future thanks to the new platforms for oligonucleotide formulation and enhanced delivery that are being continuously developed [20,41]. Since antisense therapy is a mutation-targeted treatment, with the target defects largely involving ‘private’ mutations, in the sense that they affect only an individual or his family members, it can be faced as a model of personalized medicine. This kind of approach to treat rare diseases is usually costly and rarely scalable. In this case, the type of technology used proved to have potential application in extremely rare cases, such as the subject of this project. And the question remaining would be an ethical one: Why not carry on when there are potential means to proceed? Rare diseases can be profitable if scalable treatments exist. Neglecting unique patients and providing them with palliative care is certainly easier than a time-consuming, multi-step, expensive approach. The current regulatory process for drug development involves clinical trials and evaluation of clinical outcomes, which is quite difficult to implement for antisense specific oligonucleotide drugs. Therefore, efforts are currently being made to allow AO chemistries to be treated as a single drug class, which will dramatically accelerate progress in the development of antisense drug therapies for RNA mis-splicing diseases [24,42]. Perhaps the future will provide better care and more precise means of diagnostics for rare diseases, which will save effort, money and the suffering of patients with rare genotypes.

## 5. Conclusions

In conclusion, this work constitutes a proof of principle for the in vitro correction of a *CSTB* gene defect causing ULD with a mutation-specific antisense approach. Further studies are needed to assess the therapeutic potential of AOs, including how they could be delivered to specific organs and tissues without limitations. Still, the insights obtained from this study make mutation-based therapy a clear candidate for personalized treatment of ULD patients, encouraging similar investigations of other genetic diseases.

## Figures and Tables

**Figure 1:**
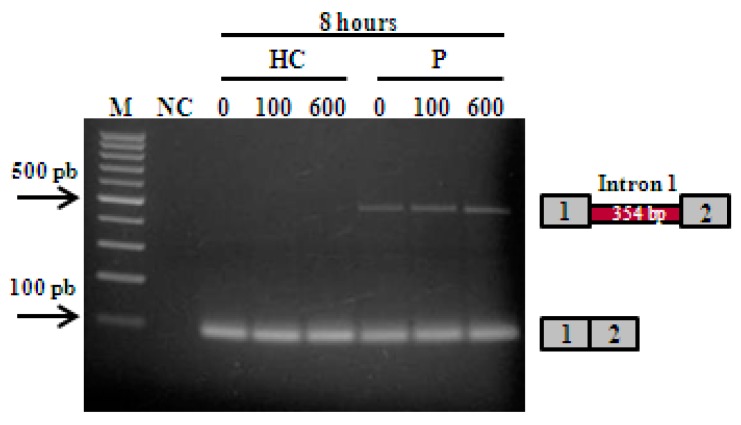
Transcriptional profile obtained after reverse transcription (RT)-PCR amplification of RNA extracted from healthy control (HC) and patient (P) fibroblasts, untreated and treated with two different concentrations of cycloheximide (100 and 600 µg/mL) for 8 h. Agarose gel electrophoresis showed one band for control fibroblasts and two for patient. The patient pattern revealed an upper band (451 bp) corresponding to an aberrant spliced transcript with the insertion of 354 bp of intron 1 (between exons 1 and 2), and a lower weight band (87 bp) for a transcript with exons 1 and 2, with the synonymous nucleotide change c.66G>A in the last base of exon 1. The control profile showed only a correct spliced product composed of exons 1 and 2. M—molecular marker; NC—negative control; HC—healthy control; P—patient.

**Figure 2:**
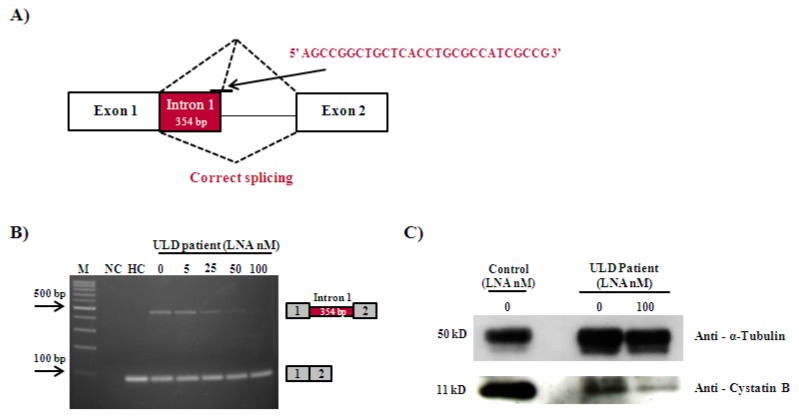
Antisense locked nucleic acid (LNA) therapeutic approach in Unverricht-Lundborg disease (ULD), patient-derived fibroblasts. (**A**) Schematic representation of the splicing downregulation observed in the presence of the c.66G>A *CSTB* mutation. The sequence of the LNA complementary to the cryptic donor site activated by the mutation and designed in order to block the recognition of the intronic alternative 5′ss in fibroblasts from the patient is shown. (**B**) Transcriptional profile obtained for HC and patient fibroblasts untreated (0 nM) and treated with quantities between 5 to 100 nM of LNA oligonucleotide. The RT-PCR analysis showed the disappearance of the aberrantly spliced transcript (451 bp) when cells were treated with 100 nM of the LNA oligonucleotide. Correctly spliced mRNA was obtained 24 h after transfection in a dose-dependent manner. (**C**) CSTB protein expression in control and patient fibroblasts untreated (0 nM) and treated with 100 nM of the LNA. The α-tubulin protein was used as loading control. M—molecular marker; NC—negative control; HC—healthy control.
